# Overweight and obesity in urban Africa: A problem of the rich or the poor?

**DOI:** 10.1186/1471-2458-9-465

**Published:** 2009-12-15

**Authors:** Abdhalah K Ziraba, Jean C Fotso, Rhoune Ochako

**Affiliations:** 1 African Population and Health Research Center (APHRC), PO Box 10787, 00100 Nairobi, Kenya; 2 Department of Epidemiology and Population Health, Centre for Population Studies. London School of Hygiene and Tropical Medicine, 49-51 Bedford Square, London, WC1B 3DP, UK

## Abstract

**Background:**

Obesity is a well recognized risk factor for various chronic diseases such as cardiovascular diseases, hypertension, and type 2 diabetes mellitus. The aim of this study was to shed light on the patterns of overweight and obesity in sub-Saharan Africa, with special interest in differences between the urban poor and the urban non-poor. The specific goals were to describe trends in overweight and obesity among urban women; and examine how these trends vary by education and household wealth.

**Methods:**

The paper used Demographic and Health Surveys data from seven African countries where two surveys had been carried out with an interval of at least 10 years between them. Among the countries studied, the earliest survey took place in 1992 and the latest in 2005. The dependent variable was body mass index coded as: Not overweight/obese; Overweight; Obese. The key covariates were *time lapse *between the two surveys; woman's education; and household wealth. Control variables included working status, age, marital status, parity, and country. Multivariate ordered logistic regression in the context of the partial proportional odds model was used.

**Results:**

Descriptive results showed that the prevalence of urban overweight/obesity increased by nearly 35% during the period covered. The increase was higher among the poorest (+50%) than among the richest (+7%). Importantly, there was an increase of 45-50% among the non-educated and primary-educated women, compared to a drop of 10% among women with secondary education or higher. In the multivariate analysis, the odds ratio of the variable *time lapse *was 1.05 (p < 0.01), indicating that the prevalence of overweight/obesity increased by about 5% per year on average in the countries in the study. While the rate of change in urban overweight/obesity did not significantly differ between the poor and the rich, it was substantially higher among the non-educated women than among their educated counterparts.

**Conclusion:**

Overweight and obesity are on the rise in Africa and might take epidemic proportions in the near future. Like several other public health challenges, overweight and obesity should be tackled and prevented early as envisioned in the WHO Global strategy on diet, physical activity and health.

## Background

As the developed world grapples with a proportionately high burden of non-communicable diseases (in this paper the term non-communicable diseases is used interchangeably with chronic diseases) developing countries and countries undergoing socioeconomic transition are experiencing a mixed epidemic of non-communicable and communicable diseases [1]. In many developing countries, research and investment in health has been mainly devoted to infectious diseases, despite the growing need to address chronic diseases with more effort and resources [2]. Deaths from infectious diseases, maternal and perinatal conditions, and nutritional deficiencies combined are projected to decline by 3% over the next 10 years, while at the same time deaths due to chronic diseases are projected to increase by 17% [2]. As a result, it is estimated that of the projected 64 million deaths worldwide in 2015, 41 million (64%) will result from chronic diseases - unless urgent action is taken [2]. Obesity is a well recognized risk factor for various chronic health problems such as cardiovascular diseases, hypertension, stroke, type 2 diabetes mellitus, osteoarthritis and certain cancers [3,4]. These conditions not only lead to reduced quality of life given their protracted nature, they also lead to premature death. Once associated only with high income countries, overweight and obesity are now also prevalent in low and middle income countries [2,5].

Over the past several decades, the prevalence of obesity has been increasing both in developed and developing countries, and more noticeably in urban areas [6-8]. It is currently estimated that as much as 20-50% of urban populations in Africa are classified as either overweight or obese [9,10], and that by 2025 three quarters of the obese population worldwide will be in non-industrialized countries [2]. Urbanization and socioeconomic transformation comes with increased access to energy-dense foods and less strenuous jobs resulting into many people having a positive energy balance and hence becoming overweight or obese [2,10-16]. Other factors that have been shown to be associated with a higher risk of overweight and obesity include genetic predisposition, metabolic disorders, gender and physical environmental factors among others [2,9,11-15]. While low socioeconomic economic status and poor neighborhoods have been associated with a higher prevalence of obesity and chronic diseases in developed countries [17,18], studies in Africa have demonstrated by contrast a strong positive relationship between obesity and high socioeconomic status [14,19,20]. Additionally very few studies have examined the changing dynamics of overweight/obesity and socio-economic status over time, making it difficult to assess the socioeconomic differentials in the rate of progression to overweight and obesity in urban Africa [21].

Despite being the least urbanized continent, Africa's population is becoming increasingly urban and its cities are growing at unprecedented rates. Africa's urban population was 15 percent of the total population in 1950, and is projected to exceed the fifty percent milestone by 2030 [22]. Rapid urbanization amidst poorly performing economies has resulted into a large proportion of urban residents being poor with limited access to social amenities [22,23]. In spite of rampant poverty in urban areas, access to cheap foods with a high content of fat and sugar among the urban poor is easier than among the rural population [24-26]. Indeed some studies have shown that recent migrants to cities tend to have a higher body mass index (BMI) than rural residents and those with longer urban environment exposure [27]. With increasing urbanization, there might be a shift of the obesity burden to sections of the poor urban population who may not have the knowledge or financial resources to adopt healthier lifestyles [28-30]. Overall, poverty and social exclusion are likely to increase the risks of developing a chronic disease, but more importantly the poor are also more likely to develop and die of complications of chronic diseases due to their inability to afford treatment and care [2,31].

The aim of this study is to shed light on the patterns of overweight and obesity in sub-Saharan Africa, with special interest in differences between the urban poor and the urban non-poor. The specific goals are to 1) describe changes in overweight and obesity among urban women in African countries; 2) examine how these changes vary by education and household wealth.

## Methods

### Data

This paper used publicly available data from the Demographic and Health Surveys by Measure DHS who approve and allow access to the data through their website. The surveys are designed to collect good quality, nationally representative data on demographic and health indicators and are carried out at regular intervals (usually every five years from the previous survey) in most developing countries. The main aim of the DHS is to provide the much needed data on population and health situation in developing countries. The repeat surveys help to provide indicators for monitoring goals set by countries and development agencies such as those set at International Conference on Population and Development (ICPD) in 1994 and the Millennium Development Goals (MDGs) set in 2000. We used data from all sub-Saharan African countries with a survey containing women's nutritional status conducted between 1992 and 1993 and another one carried out in 2003 or later. This criterion ensured that the estimates of changes over time, using survey-specific sample weights, were based on a period of at least ten years between the two surveys. As of July 2008, seven countries qualified for the study: Burkina Faso (1992/93; 2003), Ghana (1993; 2003), Kenya (1993; 2003), Malawi (1992; 2004); Niger (1992; 2006), Tanzania (1992; 2004) and Senegal (1992; 2005). The response rates in the DHS surveys are normally high. The lowest among the countries studied was 93% in Burkina Faso in the 1992/3 survey. In this paper the earlier dataset for each country is referred to as "survey 1" and the second as "survey 2". In the DHS, mothers of children born during the five-year period before the survey are weighed and their height measured using standard procedures. The height and weight measurements are standard across all countries where DHS are conducted. Field workers are specifically trained to take and record measurements accurately. Measurements are taken by the interviewers at the household using height boards for height and electronic weighing scales for weight. Pregnant women and those who are less than three months postpartum are excluded from the anthropometric measurements. The sample size per country is shown in "Additional file 1".

### Variables

The variables used in this study were obtained from two types of questionnaires: the individual women's and household questionnaire. The individual women's questionnaire provided information on the characteristics of the woman while the household questionnaire provided information on household possessions and amenities such as source of drinking water, toilet facilities and dwelling characteristics which were used to create a socio-economic status index, "wealth index". A scoring method was used to calculate the wealth index based on the presence of amenities. The dependent variable was the women's body mass index (BMI) defined as the weight (in kilograms) divided by the square of the height (in meters), also expressed as (kg/m^2^). For the purpose of this analysis, the variable was re-coded as tricotomous: Not overweight/obese; overweight; obese. Using the conventional cut-off points, overweight was defined as BMI between 25 and 30; and obesity as BMI greater or equal to 30 [32]. The key covariate of interest was the time (in years) between survey 1 and survey 2 (referred to as "time lapse") which allowed the estimation of change over time in overweight and obesity. The two other central covariates were woman's education and a household wealth index. In the absence of data on income or expenditures, the wealth index has been shown to be a robust measure of household wealth in developing countries [33]. Finally, the control variables included working status, age, marital status, parity, and country.

### Statistical method

Multivariate ordered logistic regressions were fitted in the context of the partial proportional odds model. This model was chosen since the dependent variable was a three-category ordinal outcome. Using multinomial regression would mean that the information conveyed by the ordered nature of the outcome variable is discarded. In addition, not treating the variable as ordered, would potentially lead to loss of efficiency [34]. The partial proportional odds model is a special case of the generalized ordered logit model that is less restrictive than the proportional odds model. Though proportional odds models are suited for the analysis of ordinal response variables, a critical assumption is that of *parallel slopes*. This assumption was assessed in preliminary analyses, using Brant test. The test showed that overall, there was a violation of the parallel regression assumption (*X*^2 ^= 212.99, p-value < 0.001) on a number of covariates. Details on the statistical theory behind the partial proportional odds model can be found in several sources [35]. The STATA command gologit2 [36] was used to fit the partial proportional odds model.

## Results

### Sample Description

**Figure 1:**
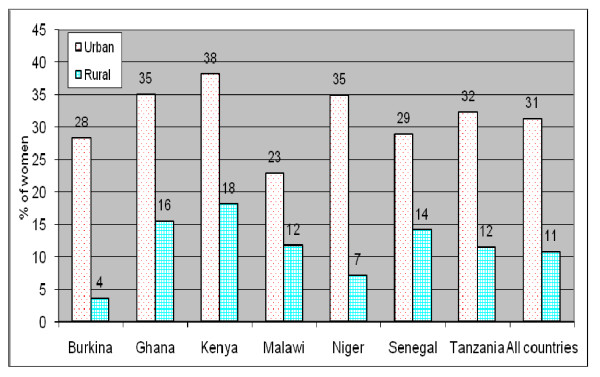
**Urban-rural differences in overweight/obesity; survey 2 (latest)**.

Figure 1 shows the prevalence of overweight/obesity in the selected countries individually and the pooled data, based on the latest surveys (survey 2). To a large extent, overweight/obesity was concentrated in urban areas. In the pooled dataset, urban women were almost three times more likely to be overweight or obese compared to their rural counterparts. The urban to rural ratio in overweight/obesity varies from about 2 (in Malawi, Senegal, Kenya and Ghana), and 3 (in Tanzania) to as high as 5 in Niger and 8 in Burkina Faso. The prevalence in overweight and obesity in urban areas ranges from about 23% in Malawi to 35% in Niger and Ghana and 38% in Kenya. From here onwards, the analyses were based on the pooled (survey 1 and survey 2) urban sample which comprises 19,992 women as described in Table 1.

**Table 1:** Description of the Urban Sample ^1^

*Variables*	
Time lapse *(between survey 1 and survey 2)*	
Mean (Standard deviation)	11.6 (1.5)
	Percentage
Education	(%)
None	25.7
Primary	40.1
Secondary+	34.2
Working status	
Not working	44.2
Working	55.9
Age	
15-19	20.7
20-24	21.9
25-29	19.4
30-34	14.5
35+	23.6
Marital status	
Never married	29.8
Currently married	60.6
Other	9.6
Parity	
<2	45.5
2-3	25.5
4-5	14.6
6+	14.4
Country	
Burkina Faso	16.4
Ghana	16.0
Kenya	12.4
Malawi	10.4
Niger	7.3
Senegal	17.4
Tanzania	20.2
N	19,992

**Note**: Household wealth is not shown; ^1^Based on weighted percentage

As can be seen from Table 1, there was an average of 11.6 years between survey 1 and survey 2. About a quarter of women had no education whereas about 40% had primary education and 34% had secondary or higher education. Slightly more than half (nearly 56%) were working, and a majority (60.6%) were married. Each of the age groups 15-19, 20-24 and 25-29 comprised almost 20% of the total sample. Most women (45.5%) had less than two children ever born and a quarter had two or three children. Niger (7.3% of the total sample) and to a lesser extent Malawi (10.4%) had the lowest sample size, whereas Tanzania (20.2%) had the largest sample.

### Descriptive analysis

**Figure 2:**
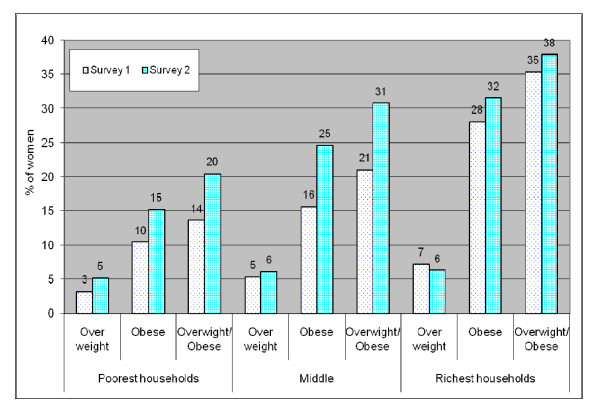
**Trends in urban overweight and obesity by household wealth**.

**Figure 3:**
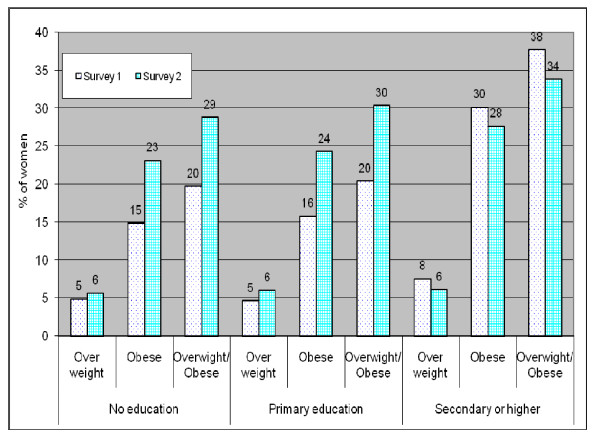
**Trends in urban overweight and obesity by education**.

Table 2 shows that the prevalence of urban obesity markedly went up by nearly 42% (from 17.9% to 25.4%) between survey 1 and survey 2. Over the same period, the prevalence of overweight rose by 14.5%, and the prevalence of overweight/obesity increased by 35.5%. Figure 2 shows changes over time in overweight and obesity by household wealth. The prevalence of overweight/obesity was highest among the wealthiest in both surveys 1 and 2. It is important to note that while overweight/obesity increased by nearly 35% (from 23.2% in survey 1 to 31.4 in survey 2 (see Table 1), the increase was higher among the poorest (+50% from 13.7% to 20.5%) than among the richest (+7% from 35.4% to 37.9%). Educational differences in trends of overweight/obesity (see Figure 3) showed an even stronger divide. While the prevalence of overweight/obesity increased by about 45-50% among the non-educated and primary-educated women, it dropped down by close to 10% among women with secondary education or higher.

**Table 2:** Change over time in the prevalence^1 ^of overweight and obesity in urban areas of the selected countries

	Survey 1	Survey 2	Var (%)^2^
Not overweight/obese	76.8	68.6	-10.7
Overweight/obese	23.2	31.4	35.5
Overweight	5.3	6.0	14.5
Obese	17.9	25.4	41.7
Statistical significance	p = 0.000		
			
N	5,887	14,105	

^1^Based on weighted percentage

^2^Percentage of variation between survey 1 and survey 2

### Multivariate model

Table 3 shows the results of the multivariate analysis. Overall (pooled sample), the likelihood of overweight and obesity increased over time in urban areas. The odds ratio of the variable *time lapse *was 1.05 (p < 0.01), indicating that the prevalence of overweight/obesity increased by about 5% per year on average in the countries in the study.

**Table 3:** Odds ratios of ordered logistic regression on overweight and obesity in selected African countries

	(Overweight or obese) vs Not overweight/obese		Obese vs (overweight or not overweight/obese)	
		
	Odds Ratio	95% Confidence Interval	Odds Ratio	95% Confidence Interval
Time lap (Continuous)	1.05**	[1.04; 1.06]		^a^Same
Household wealth (Ref: Poorest)				
Middle	1.97 **	[1.80; 2.16 ]		Same
Richest	3.20 **	[2.92; 3.51 ]		Same
Education (Ref: None)				
Primary	1.40 **	[1.27; 1.53 ]		Same
Secondary+	1.59 **	[1.44; 1.76 ]		Same
Working status (Ref: Not working)				
Working	1.13 **	[1.05; 1.22 ]		Same
Age (Ref: 20-24)				
15-19	0.61 **	[0.53; 0.70 ]	0.50 **	[0.43; 0.59 ]
25-29	1.52 **	[1.36; 1.69 ]		Same
30-34	2.49 **	[2.19; 2.82 ]		Same
35+	3.28 **	[2.87; 3.74 ]	3.57 **	[3.12; 4.08 ]
Marital status (Ref: Current married)				
Never married	0.62 **	[0.55; 0.70 ]		Same
Other	0.91 ^†^	[0.82; 1.02 ]		Same
Parity (Ref: <2)				
2-3	1.22 **	[1.10; 1.36 ]		Same
4-5	1.26 **	[1.10; 1.43 ]		Same
6+	1.22 **	[1.05; 1.41 ]		Same
Country (Ref: Burkina)				
Ghana	1.40 **	[1.24; 1.58 ]		Same
Kenya	1.54 **	[1.37; 1.74 ]		Same
Malawi	0.79 **	[0.69; 0.91 ]		Same
Niger	1.49 **	[1.32; 1.67 ]		Same
Senegal	1.21 **	[1.07; 1.36 ]		Same
Tanzania	1.19 **	[1.05; 1.34 ]		Same

^†^p < .10;*p < .05; **p < .01

^**a**^Estimates for (Overweight or obese) vs Not overweight/obese are the same as for Obese vs (overweight or not overweight/obese).

All three socioeconomic variables included in the analysis were strongly associated with overweight/obesity. Women from the richest households were more than three times as likely as their counterparts from the poorest households to be overweight or obese (p < 0.01); women with secondary or higher education were about 60% more likely to be overweight or obese compared to their counterparts with no education (p < 0.01); and working women were more than 13% more likely to be overweight or obesity as compared to their counterparts who were not working (p < 0.01). With regard to demographic covariates, Table 3 shows that urban overweight/obesity increases with age (p < 0.01), and to a lesser degree, with parity (though women with six or more children were less likely than those with 4-5 children to be overweight or obese). Currently married women were more likely to be overweight or obese than those who had never been married (p < 0.01). Finally, country differences in the prevalence of urban overweight/obesity depicted in Figure 1 were apparent in the multivariate results. Malawi had the lowest prevalence (Odds Ratio of 0.79), followed by Burkina Faso (OR of 1.00). At the other end of the scale, Kenya (OR of 1.54) and Niger (1.49) recorded the highest risk of urban overweight/obesity.

### Interactive model

The interaction variables between the variable *time lapse*, and household wealth and education are displayed in Table 4. The interaction between the variable *time lapse *and richest household was low and did not reach statistical significance at the level of 10%, indicating that the rate of change in urban overweight/obesity did not significantly differ between the poor and the richest. Further calculations derived from the ORs in Table 3 (not shown) suggest that the rate of increase in overweight/obesity was 3.9% per year among the poorest, and 4.5% per year among the richest but statistically not different. The interaction between secondary or higher education and *time lapse *was lower than one and statistically significant, indicating that the rate of increase in urban overweight/obesity was substantially higher among the non-educated women than their educated counterparts. This result was in line with the descriptive findings. Further calculations (not shown) revealed a rate of increase of 5.4% among the non-educated and only 2.3% among the well educated women.

**Table 4:** Odds ratios of ordered logistic regression on the interactions between "Time lapse" and household wealth and education in selected African countries

	Interaction between Household wealth and Time lap		Interaction between Education and Time lap	
		
	Odds Ratio	95% Confidence Interval	Odds Ratio	95% Confidence Interval
Time lap (Continuous)	1.039 **	[1.02; 1.05 ]	1.05 **	[1.04; 1.07 ]
Household wealth - Time lap Interaction				
Middle - Time	1.018 *	[1.00; 1.04 ]		
Richest - Time	1.005	[0.99; 1.02]		
Education - Time lap Interaction				
Primary - Time			1.00	[0.99; 1.02 ]
Secondary+ - Time			0.97 **	[0.95; 0.99 ]

^†^p < .10;*p < .05; **p < .01

**Note**: Estimates for (Overweight or obese) vs Not overweight/obese are the same as for Obese vs (overweight or not overweight/obese)

Interaction between country and time lapse is shown in Table 5. Burkina Faso (the reference category), Ghana, Kenya and Tanzania had comparable rate of change over time, while the three remaining countries recorded significantly lower rate of increase in overweight/obesity.

**Table 5:** Odds ratios of ordered logistic regression on the interactions between "Time lap" and country

Variable	Odds Ratio	95% Confidence Interval
Time lap (Continuous)	1.071 **	[1.05; 1.09]
Country - Time lap Interaction		
Ghana - Time	0.999	[0.97; 1.03]
Kenya - Time	1.005	[0.97; 1.04]
Malawi - Time	0.964 **	[0.94; 0.99]
Niger - Time	0.969 **	[0.95; 0.99]
Senegal - Time	0.961 **	[0.94; 0.98]
Tanzania - Time	0.985	[0.96; 1.01]

†p < .10; *p < .05; **p < .01. Note: Estimates for (Overweight or obese) vs Not overweight/obese are the same as for Obese vs (overweight or not overweight/obese).

## Discussion

This paper examined changes over time in the prevalence of overweight and obesity among urban adult women in seven sub-Saharan African countries; and investigated the extent to which these changes vary by household wealth and education. Our data for the latest survey showed that on average 31.4% of the women were overweight or obese, with prevalence as high as 38% in Kenya; 35% in Niger and Ghana; and 32% in Tanzania; lower values were observed in Malawi (23%), Burkina and Senegal (28-29%). We speculate that this differential by country could be related to the level of economic development and level of urbanisation, although this is unlikely to be the case with Niger.

Our descriptive results on trends over time indicate that overweight/obesity increased by nearly 35.5% between survey 1 and survey 2, as a result of a steep rise in the prevalence of obesity (+42%) and a smaller increase in overweight (+14.5%). The multivariate results further showed that the prevalence of overweight/obesity increased by an average of nearly 5% per year. This finding reinforces the observation that obesity is on the increase in urban areas of Africa, and lends support to the WHO warning on an impending dual epidemic of communicable and non-communicable diseases in African in the near future [2]. In line with other studies [6,7,21], our findings showed that women of higher socio-economic status (proxied by household wealth and women's education) were more likely to be overweight or obese than their poorer counterparts. Similarly women who were engaged in income generating activities (working) were more likely to be overweight or obese. This pattern might be related to changing nutritional and lifestyle trends, with urban populations consuming more refined and energy-dense foods and having fewer opportunities for physical activities.

A striking finding from this study was the increase in obesity among women of lower socio-economic status. The speed of increase in overweight/obesity emerged in the descriptive analysis to be higher among the poorest group (+50%) than the richest group (+7%), although the difference was not apparent in the multivariate results (interactive model). The household wealth measure used in this paper may not be appropriate for detecting changes in household wealth over time as household assets and characteristics may not change in the short term secondary to changes in household income. Further research using other measures of wealth (e.g. income or expenditures) might improve our understanding of changes in overweight and obesity over time by economic differentials.

If educational attainment was to be used as a proxy for socioeconomic status of individuals, both our descriptive and multivariate analyses revealed that the changes in the proportion of overweight/obese individuals in the two surveys were highest amongst the poorest segment of the population studied. The descriptive results showed that while the prevalence of overweight/obesity went up by nearly 46% among the non-educated women, it dropped by about 10% among women educated at the level of secondary level or higher. This pattern was confirmed by the multivariate results, with estimated annual rate of increase of 5.4% among the non-educated, compared to 2.3% among those with secondary or higher education. This finding suggests that over time, poor urban residents will become as affected as their wealthier counterparts or even worse off, as is now the case in the developed world. In the context of rapid urbanization in Africa, primarily driven by rural-urban migration [37], the new migrants have been shown to adapt to new urban life styles which ultimately predispose them to becoming obese even though their socioeconomic status might be lower than that for long term residents [38,39]. Further, the health consequences of big body size may not be known to the poor; and indeed some studies have shown that in some sections of African populations, obesity may be positively regarded as a symbol of high social status [40,41]. This study also found that young and unmarried women were less likely to be obese, a result that differs from that reported in another study which showed that younger women were more likely to be obese than older women [42]. Our result might be explained by the fact that single women unlike their married counterparts are less likely to be multiparous which is associated with higher risk of obesity [43].

## Limitations of the study

The study has three main limitations. First, the data used is cross sectional, whereas the ideal situation would have been to track individuals over time and ascertain changes in body mass index and associated risk factors. Second, the female population used in this study may not be representative of the entire adult female population, given that the anthropometric measurements in the DHS are restricted to women who had given birth in the five years preceding the survey. Lastly, the lack of a uniform definition of what constitutes an urban and rural area poses a challenge in making comparisons and generalizations about observations across countries. The DHS uses the official country specific designations. More often than not the classifications vary across countries taking into account, to varying degrees, the following attributes; population density, administrative function, availability of social amenities and physical infrastructure such as hospitals, post office, schools, and markets. These differences pose a challenge in the interpretation of results from multi-country studies that attempt to make rural-urban distinctions and comparisons as the classifications may not be accurate enough to allow for this [44,45].

## Conclusion

Results from this study can be considered as an early warning that overweight and obesity, as a disease in its own right and a risk factor for many other diseases is on the increase among urban populations of sub-Saharan African, with a higher increase among the urban poor. The results also suggest that in the near future, obesity among urban women might take epidemic proportions in developing countries. It should be noted however that the wealthier and more educated individuals are still the most affected by obesity and overweight. Given the chronic nature of most diseases associated with obesity and by extension the huge cost of treatment, the prospects look grim for the already under-funded and ill equipped African health care systems to deal with a new epidemic alongside existing ones such as HIV/AIDS, tuberculosis, and malaria. As rightly pointed out in a WHO report, African countries cannot afford to say "*we must tackle the other diseases first; we are poor nations, we cannot afford to deal with chronic diseases*" [2]. Like several other public health challenges, overweight and obesity should be tackled and prevented early before it gets out of hand as envisioned in the WHO Global strategy on diet, physical activity and health, ensuring that people have access to healthy diets and get involved in physical activities [11]. Unfortunately prevention efforts are yet to be embraced on a large scale in Africa as it has been in the developed world, and near absence of these in the global Millennium Development Goal (MDG) does not help the situation [46].

## Competing interests

The authors declare that they have no competing interests.

## Authors' contributions

AKZ participated in the conception of the idea of this manuscript, conducted most of the literature review and writing the background section and writing the discussion sections. He also participated in the interpretation of results. JCF framed the research question investigated, conducted the data analysis, contributed in the writing of the paper and provided the overall guidance. RO participated in the literature review and contributed in the writing of the result section. All authors read and approved the final manuscript.

## Pre-publication history

The pre-publication history for this paper can be accessed here:

http://www.biomedcentral.com/1471-2458/9/465/prepub

## Supplementary Material

Additional file 1 **Sample Size per country and urban-rural residence**. Additional file 1 contains a table that shows the sample size for each country for survey 1(earlier survey) and survey 2 (later survey) and the overall total. Click here for file
