# Supplementary material for: Overweight and obesity in urban Africa: A problem of the rich or the poor?
Source: BMC Public Health. 2009 Dec 15;9:465. doi: 10.1186/1471-2458-9-465 (PMC2803188; doi:10.1186/1471-2458-9-465)
Supplement: Additional file 1 — Sample Size per country and urban-rural residence. Additional file 1 contains a table that shows the sample size for each country for survey 1(earlier survey) and survey 2 (later survey) and the overall total. [file 1471-2458-9-465-S1.DOC]

**Additional file 1**. Sample Size per country and urban-rural residence

|  | | | | |
| --- | --- | --- | --- | --- |
|  |  | Urban | Rural | Total |
| Burkina Faso | DHS 1992/93 | 1,273 | 2,194 | 3,467 |
|  | DHS 2003 | 2,704 | 8,292 | 10,996 |
|  | Total | 3,977 | 10,486 | 14,463 |
| Ghana | DHS 1993 | 523 | 1,258 | 1,781 |
|  | DHS 2003 | 2,096 | 2,837 | 4,933 |
|  | Total | 2,619 | 4,095 | 6,714 |
| Kenya | DHS 1993 | 409 | 2,954 | 3,363 |
|  | DHS 2003 | 2,416 | 4,768 | 7,184 |
|  | Total | 2,825 | 7,722 | 10,547 |
| Malawi | DHS 1992 | 607 | 1,735 | 2,342 |
|  | DHS 2004 | 1,352 | 8,392 | 9,744 |
|  | Total | 1,959 | 10,127 | 12,086 |
| Niger | DHS 1992 | 1,280 | 2,061 | 3,341 |
|  | DHS 2006 | 1,397 | 2,550 | 3,947 |
|  | Total | 2,677 | 4,611 | 7,288 |
| Senegal | DHS 1992 | 771 | 3,742 | 4,513 |
|  | DHS 2005 | 2,291 | 6,868 | 9,159 |
|  | Total | 3,062 | 10,610 | 13,672 |
| Tanzania | DHS 1992 | 1,024 | 1,929 | 2,953 |
|  | DHS 2004 | 1,849 | 2,319 | 4,168 |
|  | Total | 2,873 | 4,248 | 7,121 |
| All countries | Survey 1 | 5,887 | 15,873 | 21,760 |
|  | Survey 2 | 14,105 | 36,026 | 50,131 |
|  | Total | 19,992 | 51,899 | 71,891 |
